# Supplementary material for: Empirical methods for controlling false positives and estimating confidence in ChIP-Seq peaks
Source: BMC Bioinformatics. 2008 Dec 5;9:523. doi: 10.1186/1471-2105-9-523 (PMC2628906; doi:10.1186/1471-2105-9-523)
Supplement: Additional File 1 — A variety of html documents from the USeq web site detailing the available applications, their best usage, output file type descriptions, command line menus, etc. [file 1471-2105-9-523-S1.zip › USeqUserGuides/JavaDoc.zip!/JavaDoc/util/bio/parsers/QualityFileParser.html]

QualityFileParser


|  |  |  |
| --- | --- | --- |
| **Overview** \| **Package** \| **Class** \| **Use** \| **Tree** \| **Deprecated** \| **Index** \| **Help** | |  |
| **PREV CLASS**   **NEXT CLASS** | **FRAMES**    **NO FRAMES**     **All Classes** |  |
| SUMMARY: NESTED \| FIELD \| CONSTR \| METHOD | DETAIL: FIELD \| CONSTR \| METHOD |  |


---


## util.bio.parsers Class QualityFileParser

```
java.lang.Object
  util.bio.parsers.QualityFileParser
```

---

``` public class QualityFileParser extends java.lang.Object ```

This class will read in combine quality files associated with raw sequencing results, skips blank lines, parses off the > character.
Quals and names are index matched, ie qual[3] and names[3] refer to the same thing.

---

**Constructor Summary**

`QualityFileParser(java.lang.String file)`


| **Method Summary** | |
| --- | --- |
| `java.lang.String` | `getFileName()` |
| `java.lang.String[]` | `getNames()` |
| `java.util.HashMap` | `getQualNames()`             Returns a hashmap of the fasta name: sequence |
| `java.lang.String[]` | `getQuals()` |
| `void` | `printMFP()` |
| `void` | `printReport()` |

**Methods inherited from class java.lang.Object**

`equals, getClass, hashCode, notify, notifyAll, toString, wait, wait, wait`

**Constructor Detail**

### QualityFileParser

```
public QualityFileParser(java.lang.String file)
```


**Method Detail**

### printMFP

```
public void printMFP()
```

---


### printReport

```
public void printReport()
```

---


### getQualNames

```
public java.util.HashMap getQualNames()
```

:   Returns a hashmap of the fasta name: sequence

---


### getQuals

```
public java.lang.String[] getQuals()
```

---


### getNames

```
public java.lang.String[] getNames()
```

---


### getFileName

```
public java.lang.String getFileName()
```


---


|  |  |  |
| --- | --- | --- |
| **Overview** \| **Package** \| **Class** \| **Use** \| **Tree** \| **Deprecated** \| **Index** \| **Help** | |  |
| **PREV CLASS**   **NEXT CLASS** | **FRAMES**    **NO FRAMES**     **All Classes** |  |
| SUMMARY: NESTED \| FIELD \| CONSTR \| METHOD | DETAIL: FIELD \| CONSTR \| METHOD |  |


---
